# Perceived role of hot food in the pathogenesis of oesophageal cancer: a qualitative study in the Arsi Zone, Oromia, Central Ethiopia

**DOI:** 10.1017/jns.2020.53

**Published:** 2021-01-08

**Authors:** Haji Aman Deybasso, Kedir Teji Roba, Tefera Belachew

**Affiliations:** 1 Adama Hospital Medical College and PhD Candidate in Human Nutrition at Jimma University, Jimma, Ethiopia; 2 College of Health and Medical Sciences, Haramaya University, Harar, Ethiopia; 3 Department of Human Nutrition and Dietetics, Jimma University, Jimma, Ethiopia

**Keywords:** Awareness, Oesophageal cancer, Dietary practices, Qualitative study, Ethiopia

## Abstract

Observational studies in Ethiopia have identified a positive association between hot wheat porridge consumption and oesophageal carcinoma. However, a single dietary intake cannot be a sufficient predictor of cancer among populations that have diverse dietary practices. The present study was carried out to explore the community's perspectives on the role of hot foods in the pathogenesis of oesophageal cancer in Ethiopia. Focus group discussions were conducted from May to August 2019 among purposefully selected 112 participants. Data were collected by using open-ended questions; memo writing, audio recordings and photo pictures. All tape-recorded interviews were transcribed verbatim and inductively coded using Atlas.ti Version 7.0.71 software. Finally, the analysis was performed according to the standard thematic framework analysis techniques. The finding showed that hot foods (porridge, coffee and soup) consumption patterns were perceived as the principal dietary risk of oesophageal cancer. Cooking in unventilated rooms, monotonous cereal-based foods, poor vegetable, and fruit intake, not taking milk with porridge, eating fast, swallowing large bolus of hot porridge and exposure to carcinogens in foods were regarded as predisposing dietary practices to oesophageal carcinoma. Socio-demographic, economic and cultural backgrounds were reported as the underlying risk factors associated with oesophageal cancer. There was a strong perception within the community that oesophageal cancer is linked to several but sequentially interlinked dietary and related practices. Cumulative thermal injuries from the consumptions of hot food could be the immediate dietary risk factors associated with increased risk of oesophageal cancer.

## Background

Perception is a proxy indicator of the awareness of illness and its causes. It is generated either from the medical knowledge, experiences from illness or shared from friends, family members and the communities^(1–4)^. Perceptions about illness include multiple components such as the name of the illness, symptoms associated with the illness, causes and consequences of that illness and the extent to which the illness can be controlled^(5)^.

Oesophageal cancer (OC) is a poor prognosis cancer that has a variable geospatial incidence worldwide^(6)^. Several researchers have reported well-demarcated endemic areas with high age-standardised incidence rates from 50 to over 100 cases per 100,000 populations in Asia^(6–8)^. Certain East African countries are in EC endemic areas that extend along a north-south corridor from Sudan and Ethiopia down to South Africa^(7–9)^.

Diet and chronic diseases have complex interactions since human diets are considered as a ‘double-edged sword’ that carry both beneficial and harmful substances^(10)^. Nutrients in the food prevent cancer through inhibiting cell damage, repair, suppressing the expression of oncogenes and stimulating the immune system^(11–14)^. On the other hand, human diets increase the risk of cancer by about 20–30 %^(14)^.

Multiple studies have revealed positive associations between the consumption of hot foods^(15–17)^, animal products^(18)^, micronutrient deficiencies^(19)^, exposure to carcinogen chemicals in food^(20)^ and increased risk of oesophageal carcinoma.

Observational studies in Ethiopia have identified a positive association between consumption of hot wheat porridge and oesophageal carcinoma^(21,22)^.

However, a single dietary intake cannot be a sufficient predictor of cancer among populations that have diverse dietary practices^(23)^.

Therefore, the present study was carried out to explore the community's perspectives on the role of hot food in the pathogenesis of EC in Ethiopia. The findings from the present study will contribute evidence for the prevention of EC in high-risk areas in the country.

## Subjects and methods

### Study setting and design

The study was conducted in Arsi Zone located in Oromia National Regional State at Central Ethiopia. The zone lies between 60 45′ N to 80 58′ N and 380 32′ E to 400 50′ E. Assela is the capital town of the Zone located at 175 km from Addis Ababa (the capital city of Ethiopia). There are 26 districts, 498 rural and 58 urban kebeles (smallest administrative structures). Oromos and Muslims are the largest ethnic and religious groups, respectively^(24)^. Barley, wheat and maize are the pre-dominant cereals and among pulses, horse beans and field, peas are grown widely. Vegetables, root crops and stimulants are also grown^(25)^. A qualitative descriptive study design was employed to generate data using focus group discussions (FGD). The study was conducted from 12 May to 15 August 2019.

### Sampling procedure

The districts in Arsi Zone were stratified into low, mid and high land agro-climatic divisions. Then, two districts (Doddota, Zuway Dugda, Hetosa, and Sude, Diksis, and Lemu bilbilo) were randomly selected from each agro-climatic division. Finally, two rural kebeles from each district were randomly selected with help of district health offices and health extension workers (HEW). The participants were recruited purposefully and stratified according to their sex to facilitate for free and informal discussions. The participants provided contact information to the HEW. The research team communicated the interested participants ahead of the discussions days. The target number of participants for FGD was 8–10. Participants were recruited until saturation on the topics under the study was achieved (i.e. key points were repeated and no significant new information was emerging).

### Data collection instrument and procedures

Four experienced data collectors (moderator and note-taker) and two supervisors were recruited based on fluency in local language and academic achievements. Participants seat around a large table in a circle of chairs and given name tags to wear. The topics for discussions included perceptions on EC (definition, symptoms and the local names), dietary practices (source crops, staple diets, and place for food preparation, dinner time, source of fuel and water for food preparation and food intake modalities) and perceived relationships between diets and oesphageal cancer. Data were collected by using open-ended questions; memo writing, audio recordings and photo pictures. FGD with men and FGD with women were facilitated by male and female data collectors, respectively. The facilitators used pausing, probing and question-rephrasing techniques to clarify respondents’ ideas or questions. Redundant responses were considered saturations and removed after transcription of daily performances. New questions were added during FGD when required. Data collections were performed at preferred places by the participants (health posts and schools) by completely avoiding other persons besides the participants and data collectors. The FGD took 85 min on average.

### Data processing and analysis

Tape-recorded interviews were transcribed verbatim immediately after the group discussion into local language (Afaan Oromoo) and translated into English by a qualified professional. The transcripts were read repeatedly line-by-line to identify codes that capture major participants’ perceptions^(26)^. The transcribed data were then inductively coded by two coders. The FGD facilitator (Ou) analysed the group interview and another researcher (EM) who is an expert in qualitative analysis and did not participate in the FGD analysed the transcribed data. Finally, the primary investigator (HA) has discussed with data analyzers to make sure that the analysis of the data reflects the views of the participants. An agreement was reached through discussion where differences in analysis appeared. Methodological rigour was maintained by using common topic guide, experienced health professionals, making observations of food cooking and storage methods and combining professionals from different backgrounds. The summary of the transcripts were returned back to the participants for feedback, while the themes were presented to the field researchers in a 1-d meeting to validate the relevance and correct representation of the data.

Data were coded by using Atlas.ti Version 7.0.71 software (Scientific Software Development GmbH, Berlin). The analysis was performed according to standard thematic framework analysis techniques^(27)^.

### Ethics approval and consent to participate

The study was conducted according to the guidelines laid down in the Declaration of Helsinki and all procedures involving human subjects/patients. Ethical permission to carry out the study was obtained from the Institutions Research Board (IRB) of Jimma University. At the start of each interview, data collectors read a standardised informed consent and confidentiality statement in the local language. The participants were asked for oral consent and permission to audio-record the conversations and photo pictures. Privacy and confidentiality were maintained throughout the study by ensuring that no names were known or written.

## Results

A total of 112 participants comprising eight to ten members from twelve FGD (six with men; six with women) were conducted. The age of the participants ranged from 40 to 90 years. The majority of the participants (53.6 %) were 60 and above years. Ninety-four (83.9 %) of the participants were Oromo by ethnicity. Regarding their sex, 61 (54.5 %) of them were male. Muslims accounted for 56.3 % of the total participants. All participants were farmers from rural residents (Table 1).

**Table 1.** Socio-demographic characteristics of participants of diets and oesophageal cancer, Arsi Zone, Central Ethiopia, 2020

Variables	No. (%)
Age	
40–59 years	52 (46·4)
≥60 years	60 (53·6)
Sex	
Male	61 (54·5)
Female	51 (45·5)
Ethnicity	
Oromo	94 (83·9)
Amhara	18 (16·1)
Religion	
Muslims	63 (56·3)
Orthodox	49 (43·7)

## Themes of the study

**Fig. 1.**
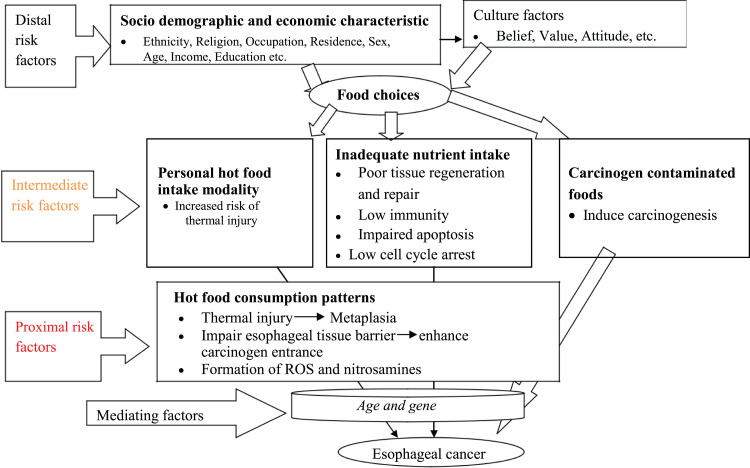
Theoretical framework of the association between diet and OC developed from the community's perspectives in Arsi Zone, Central Ethiopia, 2020.

During discussions, four main themes (awareness of OC, dietary risk factors, the influence of background variables and points of contention about the risk of OC) have emerged as the primary domains with their respective sub-themes. The perceived dietary risk factors associated with increased incidence of OC from the community's perspectives were organised into a conceptual framework as proximal, intermediate, distal dietary risk factors (Fig. 1).

### Theme one: awareness about cancer

Almost all participants heard about oesophageal cancer. Different local names (*Kaansarii Kokkee, Kaansarii Liqimsaa, Liqimsa dhoorgaa and Hudhaa)* were assigned to distinguish OC from other diseases. The word ‘Kaansarii’ is directly taken from ‘cancer’ and pronounced in Afaan Oromoo dialects, whereas ‘Kokkee, Liqimsaa, Liqimsa dhoorgaa and Hudhaa’ represent throat, oesophagus, the difficulty of swallowing and strangulation, respectively. Kaansarii Kokkee (meaning cancer of throat) and Kaansarii Liqimsaa (meaning cancer of the oesophagus) were the commonest interchangeably used local names of oesophageal cancer.

Perception regarding symptoms of OC was limited to dysphagia (difficulty of swallowing food and/or liquid). The general understandings of the discussions were reflected as

‘…*heart burn, vomiting, loss of appetite and gastric fullness are common symptoms among our communities. We don't think they are serious symptoms that related to cancer of this type (OC) and nobody informed us either. We say it is just something that goes away easily. For us, oesophageal cancer is considered when someone develops difficulty of swallowing food or liquid.*’

Participants categorised their communities into ‘higher and lower risk populations’ based on perceived disparities in dietary practices associated with increased incidences of oesophageal cancer. The reported higher risk populations were Arsi Oromo ethnic groups and/or Muslims.

### Theme two: dietary risk factors associated with OC

Heterogeneity in hot food consumption patterns was mentioned as the major difference between the high and low-risk populations (Christians). Hot foods (porridge, coffee and soup) consumption patterns were perceived as the principal foods associated with an increased risk of developing oesophageal cancer.

#### Hot porridge consumption patterns

Porridge is mainly prepared from barley, wheat and maize crops. High-risk populations (Arsi/Muslims) mentioned that porridge is consumed as a routine diet or during Ramadan (Muslims’ fasting month), on funeral and wedding days. Other ethnic/religious groups consume porridge only during Meskel holiday (finding of the true cross) or as a postnatal food for mothers who delivered a baby. Arsi/Muslims participants use a clay pot for cooking and a wooden serving bowl (Qorii) for serving porridge, while Christians cook porridge in a metal pan and use a plastic or metal bowl as a serving material (Supplementary material Additional file 1). There was a general consensus that OC is more prevalent among Arsi Oromos and/or Muslims who consume very or hot porridge as a staple diet. A 60-year-old female recounted the following: ‘…*there is no doubt about who is at risk. One of the major reasons why we believe that esophageal cancer has a strong association with frequent porridge consumption patterns is that it specifically affects Arsi Oromo and/or Muslims who frequently consume hot porridge.’* **[Female, FGD8]**

The participants supported their perception by pointing out that heartburn; vomiting, poor appetite and gastric fullness are common symptoms among populations who specifically consume hot wheat porridge. Hot wheat porridge was described as the cause of severe burn to tongue, throat and oesophagus than barley and maize porridges during consumptions. The severity of burns from such porridge was elaborated by a famous proverb that says, *gubata marqaa hubatatu arga* meaning *only a wise person understands the severity of burns from hot porridge*. For example, a male participant stated that ‘*…the risk of developing esophageal cancer might be higher particularly if someone consumes hot wheat porridge. Hot wheat porridge* is the *thickest porridge, stretchable like chewing gum, and sticks to the throat until it reaches the stomach. It* s*everely burns tongue, throat and the esophagus during swallowing. Besides, it stays hotter in the stomach for several minutes so much so that the consumer can sense steam coming up to the throat for several minutes.*’ **[Male, FGD7]**

The majority of the participants have astonishingly reported the existence of discrepancy in incidences of OC within the same family despite they consume porridge from a shared bowl. The risk was associated with differences in personal food intake modalities expressed as fast eating and swallowing of a large bolus of hot porridge. The opinions were supported by the 63-year-old participant as, ‘…*I started eating very hot porridge in a hurried manner and after two bites; I shouted water! Water! Please! You don't understand its severity unless you experienced it. Swallowing very hot porridge in a hasty manner or large bolus hot porridge is like throwing a hellfire down into your throat. Since that day, I believe that, the victims of such cancer could be a member of the family who practices fast eating and/or taking a large bolus of hot porridge.*’ **[Male, FGD5]**

#### Coffee drinking patterns

In all groups, all participants reported that they drink coffee at least three times a day. Muslim participants preferred hot milk coffee. Christian participants reported special coffee boiling round at a time (three times at a time as known as Abol, Baraka and Raja) and drinking pure coffee. Participants from Arsi/Muslims mentioned using a gourd bottle, bigger glass or plastic beakers that probably contain three to four cups of coffee as a drinking vessel in contrast to Christian participants who used small coffee cups (Supplementary material Additional file 2)**.**

Drinking hot coffee was also reported as dangerous as the consumption of hot porridge. The risk of sustaining a thermal injury to the tongue, throat and the oesophagus was, however, ascribed to the frequency, volume of the coffee drunk and drinking coffee with or after consuming hot porridge.

#### Inadequate nutrient intake

Inadequate nutrient intake was regarded as the predisposing factors of oesophageal cancer. Consumption of Animal Source Foods (ASF) was rarely practised. Participants reported selling eggs for getting additional income, while the consumption of milk and milk products restricted to those who possess cows. It was a common perception that not drinking cold milk with or after consumption of hot porridge could be a predisposing factor to oesophageal cancer. The participants supported their observations by referring to the traditional rule that orders *eat porridge with the right hand and grasp a glass of milk with a left hand.* The general discussions were presented as follows:

‘…*cancer that causes difficulty of swallowing foods (OC) was not as common as these days because our ancestors used to eat porridge with the right hand and grasp a glass of milk with a left hand. Milk has very special functions both as nutrient and in neutralizing porridge temperature. It is also viscous than water and pushes stacked porridge to the tube that passes food.’*

Almost all members from the high-risk population reported that their primary diets consist of cereal-based foods (bread of various types), ‘Buddeenaa’ (Afaan Oromoo word for fermented pancake or ‘injera’ in Amharic). Besides, the majority of the participants reported low intake of vegetables and fruits because of unavailability or inaccessibility due to higher costs. A female participant had the following to say, ‘…*frequent consumption of similar foods for a prolonged time is not good for human health. Legumes, vegetables and fruits may provide preventive ingredients against cancer.’* [**Female, FGD8]**

#### Carcinogens in foods

Some of the participants believed that preparing foods in unventilated rooms, adulterants in oil and butter may augment the risk of developing oesophageal cancer. A 45-year-old participant shared his observation as, ‘…*porridge prepared with poor quality butter has a very bad smell, causes gastric discomfort and hotter during consumption. That is why repeated epigastric burn and dyspepsia are common complaints among our populations. After several observations of such events, I had conducted a kind of experiment to see if there is any difference between butter produced at home and the one bought from the market. I melted both of them and the former (homemade butter) had the same volume (one glass) and the second (market butter) turned half a glass. The market butter had very thick sediment at the bottom, impurities at the middle, and foamy substances on the top. It is worrying and shocking. I believe now, unwanted chemicals in foods may cause cancer that prevents swallowing of foods and liquids.’* **[Male, FGD4]**

All members unwaveringly reported the consumptions of chemically treated crops disregarding the warning from the professionals. They did not practice safety precautions while storing and applying chemicals to the crops. They believed that washing containers with water can remove chemicals completely. The disappearance of the chemical's odour was considered cleanliness. Chemical containers such as fertilizer sacks were reported used for storing foods, grains, powder, and for transporting vegetables. Empty plastic chemical containers were used for storing oil, salt, coffee and fetching water for washing and drinking (Supplementary material Additional file 3). One of the participants stated the following to corroborate this idea, ‘*…we do not usually follow the orders provided by the professionals. We consume chemically treated crops within 15 days or less than that. It may contain chemicals that may cause cancer of the oesophagus.’* **[Male, FGD7]**

### Theme three: background variables and oesophageal cancer

Participants unequivocally reported that socio-demographic, economic and cultural factors could be linked with increased risk of oesophageal cancer. The majority of the participants reported shifting in dietary practices has taken place over the past 40 years from more nutritious to poor quality foods due to low socioeconomic status. Low income was associated with a decline in family's food diversity, low intakes of fruit and vegetables. Cultural preference was reported as a driving factor for habitual consumption of hot porridge. Receiving food aid was accounted for by the participants from low land agro-climatic areas. Tooth loss due to aging was an additional reason for the frequent consumption of hot porridge among older individuals.

### Theme four: points of contention about EC

Small number of the participants considered OC as a random phenomenon, as a decree from God and has nothing to do with dietary practices. A few of them also assumed that alcohol protects OC because of the reported low incidence of OC among communities who drink alcohol. Furthermore, the majority of the participants perceived that swollen tonsils are the risk factor of OC than food intake. For that reasons, removal of tonsil glands (Tonsillectomy) by traditional healers *labelled as ‘local surgeons’* reported to be widely practised.

## Discussion

In the present study, participants’ perspectives on the role of dietary practices in the pathogenesis of OC were analysed to explain why some people develop OC, whiles others do not. The result showed that the perceived dietary practices and related risk factors are supposed to operate one after the other as proximal, intermediate and distal risk factors.

Therefore, thermal injury to the oesophagus from the consumptions of multiple hot foods was considered as an immediate risk factor, while personal hot food intake behaviours, inadequate nutrient intake and carcinogens in foods were perceived as intermediate-risk factors. On the other hand, socio-demographic, economic and cultural characteristics regarded as distal risk factors that influence dietary choices associated with oesophageal cancer.

Considering hot food consumption patterns as a proximal risk factor of OC sound sensible for the following reasons. In the present study, except for hot food consumption patterns, exposures to other potential risk factors of OC were reported to be similar to all participants irrespective of their ethnic, religious and socioeconomic characteristics. Secondly, the incidence of OC was reported to vary according to participants’ hot food consumption patterns. Thirdly, disproportionate hot food consumption patterns that differ in the cooking process, inputs utilised and the serving bowl was reported among high-risk populations. For instance, participants consistently associated the consumption of hot wheat porridge with increased risk of OC throughout the discussions. The perception is in agreement with research findings in Ethiopia that identified a significant positive association between consumption of hot wheat porridge and oesophageal cancer^(21,28)^. Wheat porridge was assumed to have thickness and lower heat loss than porridge made from other types of grains. Thick hot porridge may induce severe thermal injury as it may slowly move down through the oesophagus. For instance, swallowing 2.5-mm-thick solid food for a few seconds caused severe mucosal damage, increased distal oesophageal temperature and very serious painful sensation in an experimental study^(29)^.

The participants also suggested that increased risk in the incidence of OC was higher among populations who consume fat added hot porridge as their staple diets. The finding is in agreement with the studies that documented significant associations between consumption of saturated fats and oesophageal cancer^(18,30)^.

Participants from the high-risk populations reported drinking a large volume of milk coffee using a gourd bottle contrary to the lower risk populations who reported drinking pure coffee using small coffee cups. Gourd bottle contains larger volume of coffee and may also prevent heat loss to the environment than ordinary coffee cups. Besides, the volume, the temperature and presence of fat in milk coffee can elevate the beginning drinking temperature, slow cooling rate and cause synergetic severe scald burn to the oesophagus than small volume hot pure coffee^(15,31)^.

Therefore, with consideration the difference in the study design, participants’ perception of associating various hot foods with an increased risk of OC is contrary to a case-control study in Ethiopia that identified hot porridge consumption as the sole associated dietary practices with an increased risk of OC^(28)^. On the other hand, the participants’ perceptions were consistent with the findings from previous studies that found significant positive associations between hot food consumption patterns and increased risk of OC among populations living in OC endemic areas^(15,17,32)^.

In the present study, fast eating and/or swallowing of a large bolus of hot porridge while consuming porridge in a shared manner were attributed to the discrepancy in the incidences of OC within the same family. The finding is alike to the study that associated personal food intake modalities with increased risk of oesophageal cancer^(33)^. The probable assumption could be consuming porridge in a shared manner may create a sense of competition between individuals. Consequently, some individuals may perhaps eat fast and/or swallow large bolus of hot porridge without modulating the temperature through airing (drawing air into the mouth) or by mixing with saliva in the oral cavity. Such practices can induce severe thermal injury to the oesophagus. For illustration, some case report studies revealed food debris, longitudinal ulceration and gross swelling throughout the length of the oesophagus after individuals swallowed extremely hot and microwave foods in a hasty manner^(34,35)^.

Milk is a good source of calcium mineral that controls cell cycles, cell divisions and physiological death of the tumour cells^(36)^. Likewise, the majority of the participants in the present study perceived that using cold milk with or after consumption of hot porridge would offer better protection against oesophageal cancer. The perception is in conformity with the studies that found the inverse associations between milk consumption, dietary calcium intake and risk of oesophageal cancer.^(37,38)^

Consumption of hot foods at 43°C and above can alter tissue structures to cancer cells^(39)^. Furthermore, chronic tissue irritations from high-temperature foods can instigate the formation of endogenous reactive oxygen species (ROS), nitrosamines and impair tissue barriers to carcinogens^(40–44)^. However, oesophagus has a tremendous ability to recover from insults if provided with adequate nutrients^(45)^.

In the present study, wheat, maize and barley were listed as the main source crops for porridge preparation. Beside the thermal injury, the milling process can devoid wheat porridge of very essential vitamins and minerals that protect against SCC of the oesophagus^(46)^. Additionally, poor nutrient content and elevated Prostaglandin E2 (PGE2) concentrations may expose maize porridge consumers to increased risk of developing oesophageal caner^(47)^.

Inadequate intake of nutrients, legumes and vegetables were perceived as predisposing risk factors for the frequent occurrence of OC in the study areas. The perception is appreciably important because individuals who practice poor nutrient intakes are at greater risk of deficiencies of protective antioxidants against oesophageal cancer^(18,19,48–51)^. Furthermore, low legumes intakes can impair apoptosis, removal of carcinogens before entering the intestine and decrease the concentration of flavonoids, vitamin E, vitamin B, selenium and lignans that hinder the development of cancer cells^(52–54)^.

The majority of the participants in the present study reported cooking porridge in living rooms that have no ventilation. In addition, a significant number of participants reported consumption of adulterated heated fats (oil and butter), exposure to herbicides, pesticides and the use of chemically treated crops and chemical containers. Adulteration of different types of foods became a big challenge in Ethiopia^(55)^. Cooking foods in unventilated rooms exposes to carcinogens that are released from incomplete burning of firewood, charcoal and animal manures^(56,57)^. For example, a case-control study in Malawi revealed a significant association between polycyclic aromatic hydrocarbons and increased risk of squamous cell carcinoma^(58)^. Consumption of heated or fats and oils may expose to trans fatty acids (TFAs)^(59)^ which increase the risk of developing oesophageal carcinoma. There is no consistent finding regarding the association between herbicides, pesticides and risk of oesophageal carcinoma. However, in a study conducted in Taiwan, being a farmer was significantly associated with the increased occurrence of oesophageal cancer^(60)^.

Age of a person is one of the non-modifiable background variables and the most exhaustively studied risk factors associated with cancer. The majority of the participants agreed that OC is common among elderly populations. The perception is in line with numerous studies that witnessed OC as a disease of elderly people^(61–63)^. The association between age and OC is plausible because a person may be exposed to cumulative effects of carcinogen materials throughout his/her life which changes humoral and/or hormonal systems that increase the susceptibility to neoplastic transformation^(64)^. Similarly, a 75-year-old member mentioned, ‘*… my perception is, cancer is like drops of water that fill a very big barrel over longer durations. It could be what might have been started during early childhood and manifests when someone gets older. It is the result of gradual buildup that appears when the body loses strength.*’

On the other hand, they reported uneven incidences of OC within the same family that shares similar dietary practices. The reason could be, besides personal food intake behaviour, incidences and deaths from OC are to some extent attributable to differences in genetic susceptibility and variability in DNA repair pathways^(65–67)^.

This is the first study that identified the community's perspectives regarding the role of dietary practices in the pathogenesis of OC among populations living in the OC endemic area in Ethiopia. The finding has wider practical implications as Ethiopia currently is experiencing an increased level of cancer cases, and circumstantial reports on food safety concerns. The issues mentioned by the discussants deserve consideration in the national food and nutrition strategy. It will also give clues for further investigation on the possible link between dietary intake, food safety and oesophageal cancer.

### Strength and limitations of the study

It was the first study in Arsi Zone which is known as an OC endemic area in Africa's OC hot spot corridor. As it is a qualitative study, it will also provide locally relevant baseline indicators that could be used for designing preventive interventions and management of OC in the study area.

Although we used a rigorous analysis approach, the present study has some limitations. These are, the finding cannot be generalised to all populations because of purposive sampling techniques and recruiting the older age of participants.

## Conclusion

There was a strong perception within the community that OC is linked to several but sequentially interlinked dietary and related practices. Cumulative thermal injuries from hot foods consumption patterns could be the immediate dietary risk factors associated with an increased risk of oesophageal cancer.

## Supplementary material

For supplementary material accompanying this paper visit https://doi.org/10.1017/jns.2020.53. click here to view supplementary material
