# Supplementary material for: Perceived role of hot food in the pathogenesis of oesophageal cancer: a qualitative study in the Arsi Zone, Oromia, Central Ethiopia
Source: J Nutr Sci. 2021 Jan 8;10:e1. doi: 10.1017/jns.2020.53 (PMC8057510; doi:10.1017/jns.2020.53)
Supplement: Supplementary file 1 [file S2048679020000531sup001.zip › Additional_files_3 .docx]

Chemical containers used for different purposes


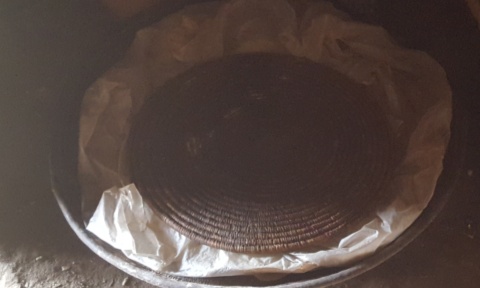

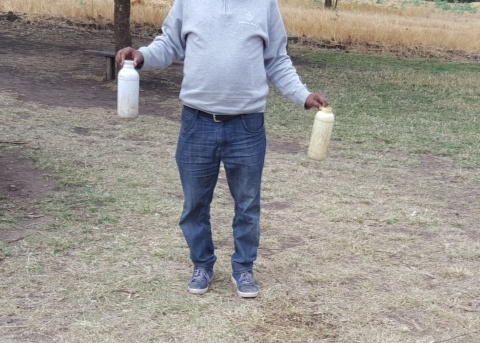


**Photo 3: Fertilizer container used for food cover (A) and herbicidal container used for drinking water, Arsi, Oromia, Central Ethiopia,2020.**
