# Supplementary material for: Perceived role of hot food in the pathogenesis of oesophageal cancer: a qualitative study in the Arsi Zone, Oromia, Central Ethiopia
Source: J Nutr Sci. 2021 Jan 8;10:e1. doi: 10.1017/jns.2020.53 (PMC8057510; doi:10.1017/jns.2020.53)
Supplement: Supplementary file 1 [file S2048679020000531sup001.zip › Additional_file_1.docx]

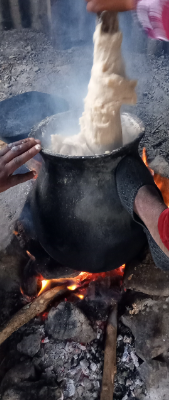

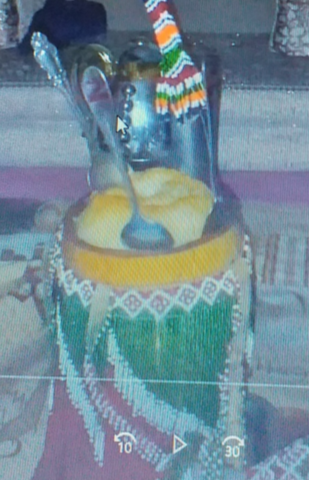

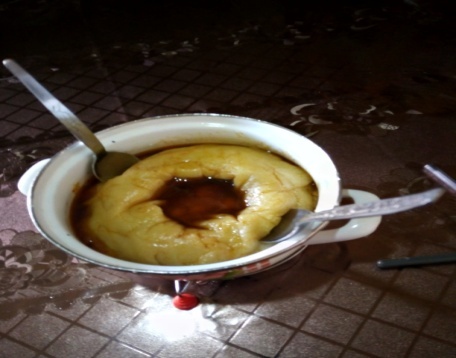


Photo1:- Cooking porridge in a clay pot (Lt), Qorii (M) and dish (Rt) for serving porridge in Arsi Zone, Oromia, Central Ethiopia, 2020.
