# Supplementary material for: Perceived role of hot food in the pathogenesis of oesophageal cancer: a qualitative study in the Arsi Zone, Oromia, Central Ethiopia
Source: J Nutr Sci. 2021 Jan 8;10:e1. doi: 10.1017/jns.2020.53 (PMC8057510; doi:10.1017/jns.2020.53)
Supplement: Supplementary file 1 [file S2048679020000531sup001.zip › Additional_file_2.docx]

Coffee drinking vessels

.


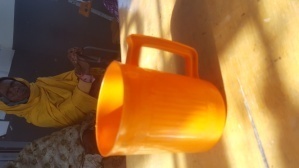


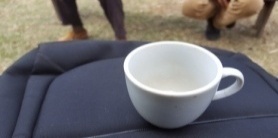

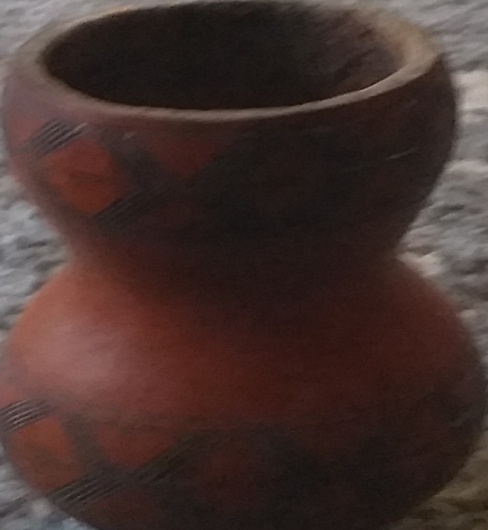


**Photo 2: Cup (Lt), plastic beaker (m) and Gourd (Rt) used for coffee drinking in Arsi Zone, Oromia, Ethiopia, 2020**
